# TrkB-enhancer facilitates functional recovery after traumatic brain injury

**DOI:** 10.1038/s41598-017-11316-8

**Published:** 2017-09-08

**Authors:** John Marshall, Joanna Szmydynger-Chodobska, Mengia S. Rioult-Pedotti, Kara Lau, Andrea T. Chin, Siva K. Reddy Kotla, Rakesh Kumar Tiwari, Keykavous Parang, Steven W. Threlkeld, Adam Chodobski

**Affiliations:** 1 Department of Molecular Pharmacology, Physiology, and Biotechnology, Brown University, Providence, RI 02912 USA; 2 Neurotrauma and Brain Barriers Research Laboratory, Department of Emergency Medicine, Alpert Medical School of Brown University, Providence, RI 02903 USA; 3 Center for Targeted Drug Delivery, Department of Biomedical & Pharmaceutical Sciences, Chapman University School of Pharmacy, Irvine, CA 92618 USA; 4 Department of Neuroscience, Regis College, Weston, MA 02493 USA

## Abstract

Brain-derived neurotrophic factor (BDNF), a key player in regulating synaptic strength and learning, is dysregulated following traumatic brain injury (TBI), suggesting that stimulation of BDNF signaling pathways may facilitate functional recovery. This study investigates whether CN2097, a peptidomimetic ligand which targets the synaptic scaffold protein, postsynaptic density protein 95, to enhance downstream signaling of tropomyosin-related kinase B, a receptor for BDNF, can improve neurological function after TBI. Moderate to severe TBI elicits neuroinflammation and c-Jun-N-terminal kinase (JNK) activation, which is associated with memory deficits. Here we demonstrate that CN2097 significantly reduces the post-traumatic synthesis of proinflammatory mediators and inhibits the post-traumatic activation of JNK in a rodent model of TBI. The recordings of field excitatory post-synaptic potentials in the hippocampal CA1 subfield demonstrate that TBI inhibits the expression of long-term potentiation (LTP) evoked by high-frequency stimulation of Schaffer collaterals, and that CN2097 attenuates this LTP impairment. Lastly, we demonstrate that CN2097 significantly improves the complex auditory processing deficits, which are impaired after injury. The multifunctionality of CN2097 strongly suggests that CN2097 could be highly efficacious in targeting complex secondary injury processes resulting from neurotrauma.

## Introduction

Traumatic brain injury (TBI), which encompasses a wide spectrum of injuries from mild to severe, has been regarded as a “silent epidemic of modern society”^1^. For patients who survive the initial trauma, morbidity and mortality are largely determined by the severity of secondary injury resulting from pathophysiological processes that lead to neuronal death^2^. The initial injury in TBI triggers glutamate release leading to hyperactivation of *N*-methyl-D-aspartate (NMDA)-type receptors (NMDARs), calcium (Ca^2+^) overload, and secondary neuronal death due to excitotoxicity^3^. However, anti-excitotoxic treatment with NMDAR antagonists has not yielded beneficial clinical effects^3^. NMDARs, which are down-regulated shortly after injury^4^, also mediate pro-survival signaling^3^, suggesting that interventions that promote NMDAR activity may be beneficial^5^. Furthermore, NMDAR activity plays a key role in cognition, being important for long-term potentiation (LTP), an activity-dependent strengthening of glutamatergic synapses^6^. Recent strategies for attenuating excitotoxic signaling have used peptidic compounds to disrupt the interaction of ionotropic glutamate receptors with the PDZ (PSD-95/Discs-large/ZO-1 homology) domains of the postsynaptic density protein (PSD)-95, an intracellular scaffold protein that couples ionotropic glutamate receptors to neuronal death pathways^7^. NMDAR-dependent cortical neuronal death is disrupted by a peptide mimicking the NR2B PDZ ligand (TAT-NR2B9c)^8^. PSD-95 also links the kainate receptor (GRIK2) with c-Jun-N-terminal kinase (JNK) activation^9^, and a peptide designed against the C-terminus of GRIK2, Tat-GluR6-9c, showed a protective effect against neuronal death induced by cerebral ischemia/reperfusion^10^.

Brain-derived neurotrophic factor (BDNF) is a key player in long-lasting increases in synaptic strength and learning^6^, and therapeutic strategies to enhance BDNF signaling after TBI could facilitate recovery^11^. BDNF binds to the tropomyosin-related kinase B (TrkB) receptor, which results in activation of downstream phosphatidylinositol 3-kinase (PI3K)-Akt, mitogen-activated protein kinase (MAPK) ERK, and PLC-γ signaling^12^, that promote neuronal survival^13^. Increased levels of glutamate activate microglia^2^, to release proinflammatory cytokines, such as tumor necrosis factor-α (TNF-α) and interleukin-1β (IL-1β)^14, 15^, to suppress BDNF-dependent signaling and LTP^16^. Tumor necrosis factor-α potentiates glutamate-mediated cell death by rapidly increasing surface expression of Ca^2+^ permeable-AMPA and NMDARs^17^, connecting neuroinflammation and excitotoxicity. The NMDAR partial agonist D-cycloserine has been shown to induce expression of BDNF, restore impaired hippocampal LTP, and improve cognitive function in a TBI model^5^, and treatment with 7,8-dihydroxyflavone (DHF), a small molecule imitating BDNF, protected hippocampal neurons^18^. In a recent study, we reported a novel PSD-95 binding peptidomimetic ligand, CN2097, that significantly up-regulated TrkB signaling^12^. CN2097 binding to PSD-95 promotes PSD-95 recruitment to TrkB to enhance BDNF-mediated PLCγ-CaMKII and PI3K-Akt signaling, but not ERK, to facilitate LTP in the hippocampus^12^. CN2097 consists of a cyclized peptide, KNYKKTEV, incorporating a β-alanine lactam side-chain linker between the valine (V) and threonine (T) residues of the PDZ-binding moiety^19^, conjugated with a disulfide bond to a polyarginine (R_7_) active-transport moiety (R_7_-CC-YK[KTE(β-Ala)]V)^19, 20^. In contrast to previously reported PSD-95 PDZ-binding peptides that disrupt the function of PSD-95^21^, CN2097 facilitates LTP^12^ and pro-survival signaling^13^.

Here we demonstrate that CN2097 has the ability to mitigate secondary injury by decreasing the production of proinflammatory mediators as well as the influx of leukocytes to the injured brain. As acute inflammation reduces LTP^22^, we evaluated the effect of CN2097 on LTP using an *in vitro* brain slice preparation, finding that CN2097 prevents deficits of LTP resulting from TBI. Auditory sensory assessment, employed to gauge the magnitude of neurological dysfunction and functional outcome after TBI, demonstrates CN2097’s ability to improve complex auditory tone-order discrimination that has been linked to language deficits in patients sustaining TBI^23^. Although the single dose of CN2097 tested in this study did not improve all therapeutic endpoints routinely evaluated in experimental TBI, taken together, our results strongly suggest that CN2097 could be highly efficacious in targeting complex secondary injury processes resulting from neurotrauma.

## Results

### CN2097 reduces post-traumatic neuroinflammation in the injured cerebral cortex

**Figure 1:**
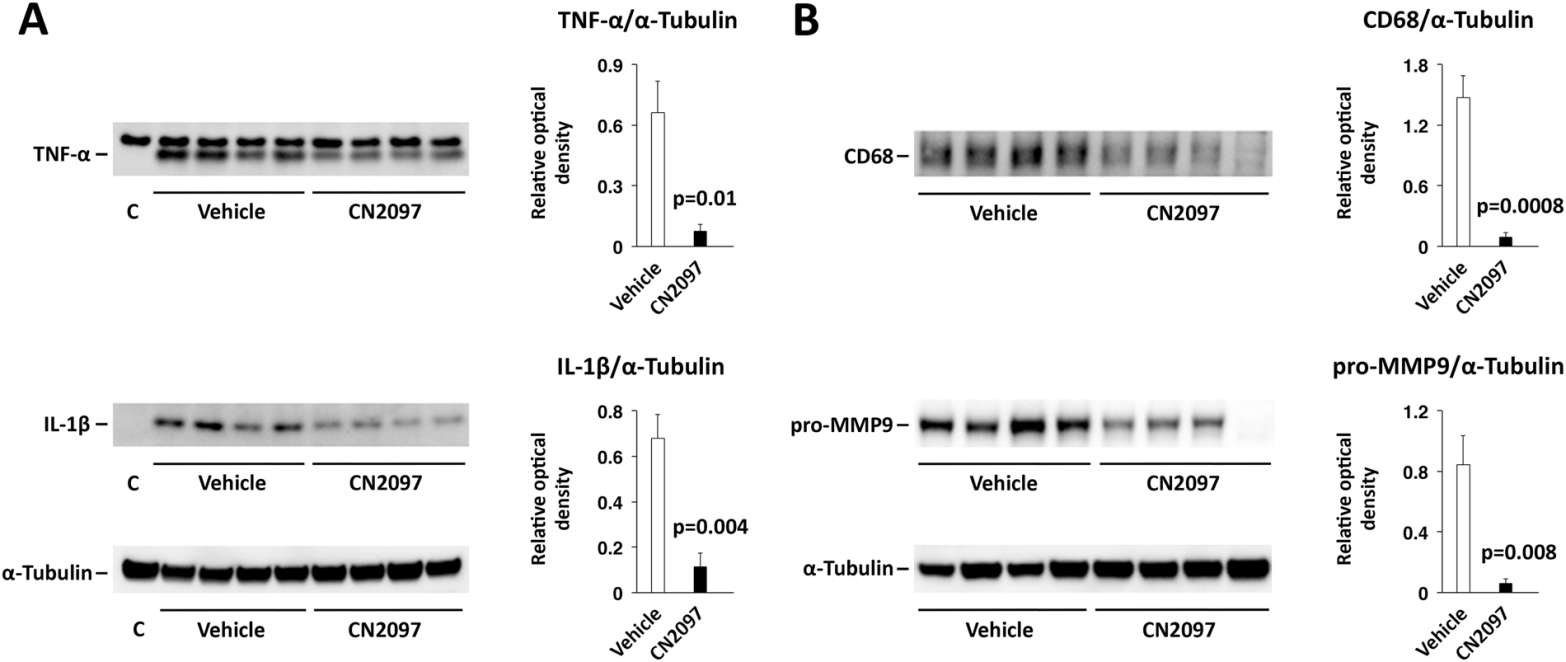
Therapeutic efficacy of CN2097 in reducing post-traumatic neuroinflammation in the injured cerebral cortex. Western blot analysis was performed on cortical samples following CCI. Rats were injected i.p. with CN2097 (10 mg/kg) or vehicle (0.9% NaCl) at 1 and 2 h post-TBI (n = 4 rats/group). Cortical samples were collected at 4 h (**A**) or 24 h (**B**) after TBI. (**A**) CN2097 significantly lowered the post-traumatic production of TNF-α and IL-1β at 4 h post-TBI. The fully processed, biologically active forms of these proinflammatory cytokines are shown. C: contralateral, uninjured cortex. (**B**) CN2097 inhibited the post-traumatic influx of monocytes to the injured cortex, as assessed by the level of expression of CD68, and reduced the synthesis of MMP9 (pro-MMP9 is shown) at 24 h post-TBI. The expression of CD68 and pro-MMP9 was undetectable in the contralateral cortex. Data represent mean ± SEM. The images shown are cropped. The full-length original images are shown in Fig. S1.

The ability of TrkB signaling to be neuroprotective in neuroinflammatory settings^24, 25^, prompted us to test if CN2097, a peptidomimetic compound enhancing downstream TrkB signaling^12^, can attenuate the brain inflammatory response to injury. Tumor necrosis factor-α and IL-1β are proinflammatory cytokines stimulating leukocyte influx, causing glial activation, and contributing to neuronal cell loss and blood–brain barrier (BBB) dysfunction^15^. In rodent models of TBI, brain concentrations of TNF-α and IL-1β increase rapidly after injury, but then return to basal levels within 24 h post-TBI^26^. Here we show that post-injury treatment with CN2097, injected at 1 and 2 h post-TBI, significantly lowered the synthesis of both TNF-α and IL-1β (Fig. 1A). As decreased production of these two proinflammatory cytokines is anticipated to dampen the inflammatory cascade, we also evaluated the magnitude of influx of leukocytes to the injured cortex. Focusing on monocytes, whose influx to the injured brain could be readily assessed by the levels of expression of CD68 at 1 d post-TBI^26^, we found that CN2097 reduces the post-traumatic influx of these inflammatory cells (Fig. 1B, upper panel). Because invading leukocytes carry and then release matrix metalloproteinases, we examined whether CN2097 would also have an effect on post-traumatic expression of matrix metalloproteinase 9 (MMP9), a metalloproteinase known to disrupt the integrity of the BBB^15^. The level of MMP9 assessed in the ipsilateral cortex at 24 h post-TBI was found to be significantly lower in CN2097-treated rats compared to vehicle-injected animals (Fig. 1B, lower panel). These results suggest that CN2097 exhibits anti-inflammatory properties by reducing the production of upstream mediators of inflammation.

### CN2097 attenuates TBI-induced hippocampal LTP deficits

**Figure 2:**
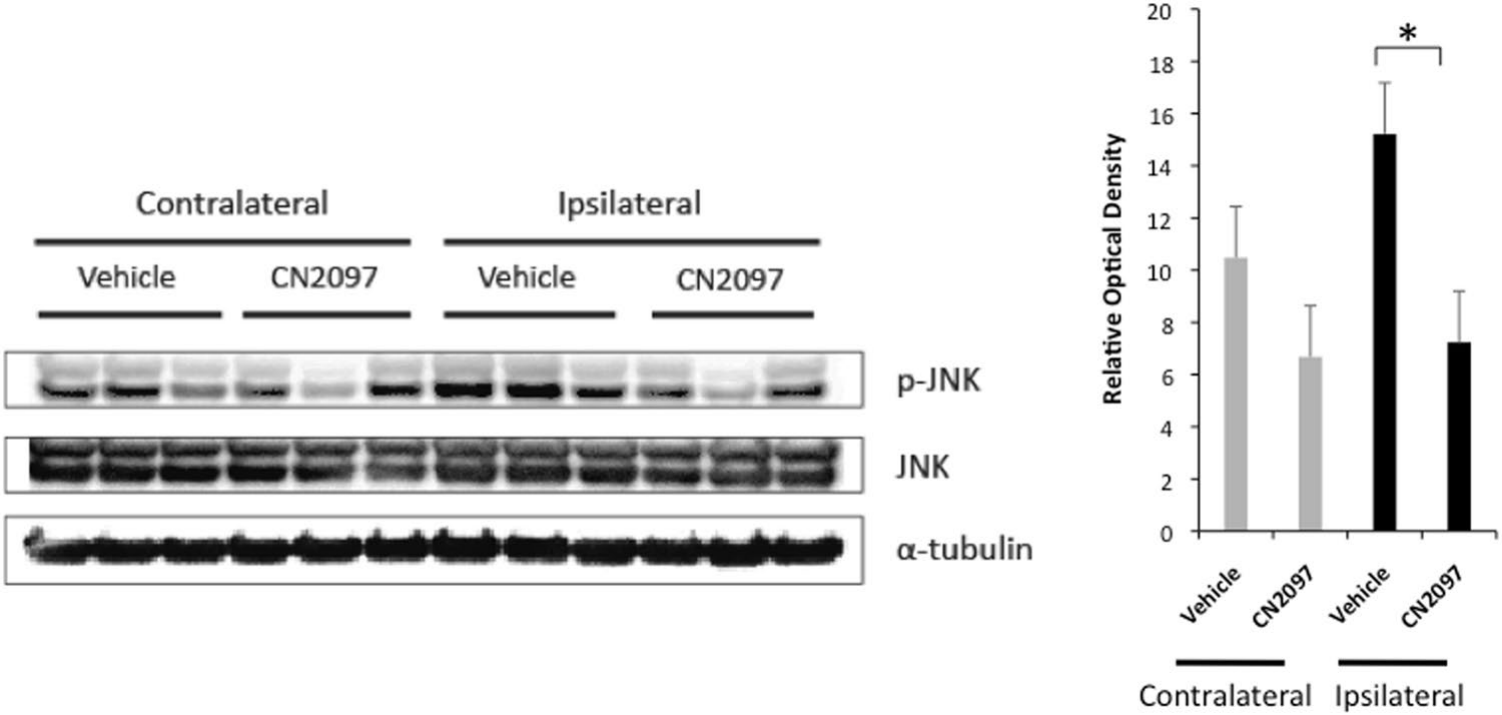
CN2097 reduces JNK activity in the injured ipsilateral hippocampus. Western blots showing that CN2097 significantly lowered the level of phosphorylation of JNK (p-JNK) in the ipsilateral hippocampus 24 h after TBI. The bar graph illustrates quantitative data of the optical density of p-JNK normalized to JNK (mean ± SEM, *p < 0.05). Animals were injected i.p. with CN2097 (10 mg/kg) or vehicle (0.9% NaCl) at 1 and 2 h post-TBI (n = 3 rats/group), and the hippocampal samples were collected at 24 h after TBI. The images shown are cropped. The full-length original images are shown in Fig. S2.

The hippocampus is highly vulnerable to neurotrauma, which leads to cognitive impairment^27^. The long-lasting increases in synaptic strength and learning in the hippocampus are highly dependent on BDNF^28^, and proinflammatory cytokines, such as IL-1β, can disrupt BDNF signaling cascades and inhibit LTP^16, 22^. It has been shown that IL-1β activates JNK, and that the inhibitory effect of lL-1β on LTP could be reversed by selective inhibition of JNK catalytic activity^29^. Three genes, *JNK1*, *JNK2*, and *JNK3*, encode the JNK proteins, and we have previously demonstrated that TBI results in a significant increase in activity of JNK1 and JNK2, whereas the activity of JNK3, which is high in both the contralateral hemisphere and in sham-injured animals, is not affected by the impact^26^. In contrast to JNK3, the activity of JNK1 and JNK2 in the contralateral hemisphere and in sham-injured rats was found to be low. When an antibody recognizing the phosphorylated forms of all three JNKs was used on immunoblots of protein extracts from the hippocampus, we found no difference in the level of JNK activation between the ipsilateral and contralateral hemispheres of vehicle-treated rats (Fig. 2), which was consistent with our previous observations^26^. In comparison, there was a significant reduction in JNK activity in the ipsilateral hippocampus in CN2097- versus vehicle-treated rats (Fig. 2). These findings suggest that CN2097 has the ability to mitigate the post-traumatic activation of JNK.

**Figure 3:**
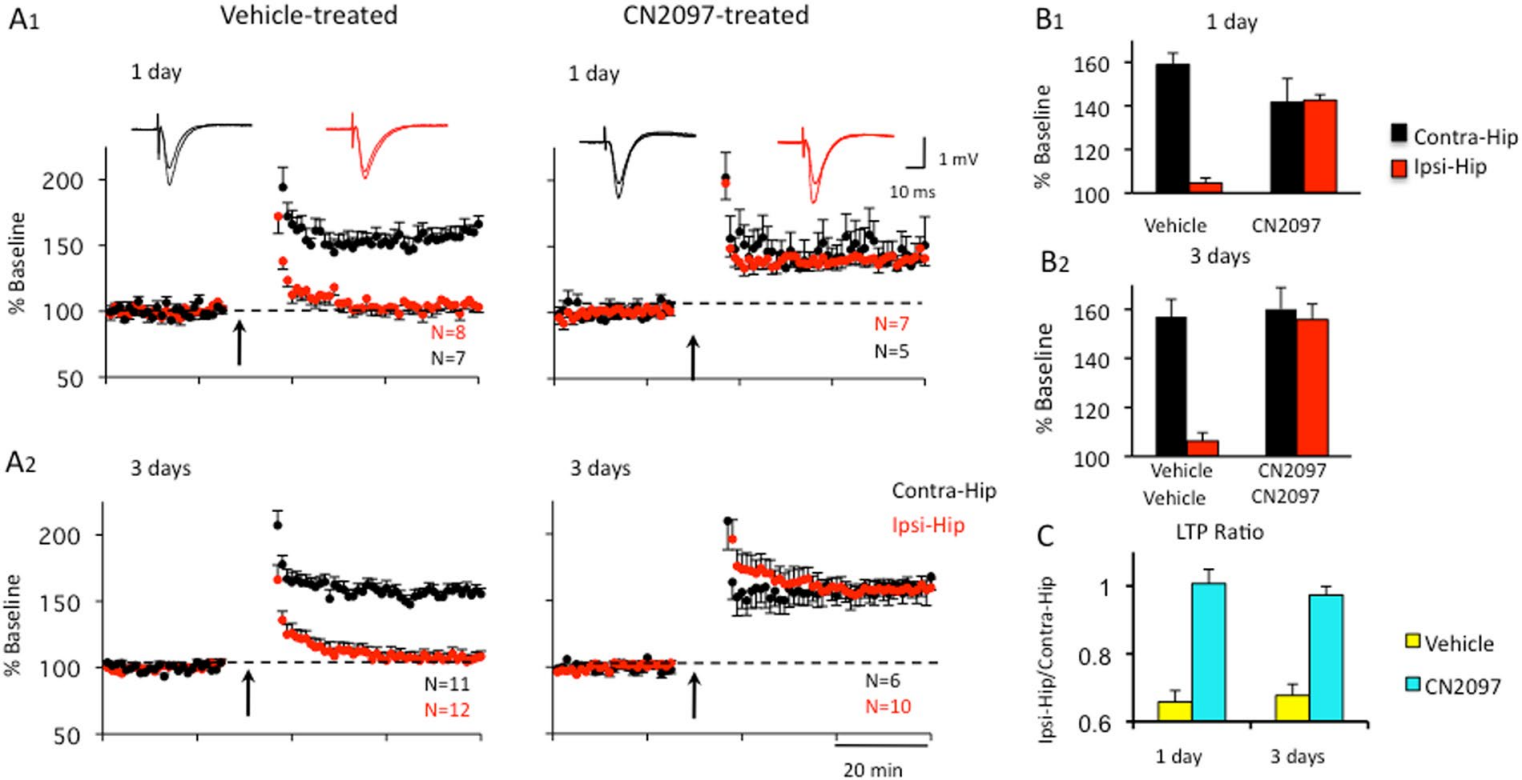
CN2097 attenuates TBI-induced hippocampal LTP deficits. Rats were injected i.p. with CN2097 (10 mg/kg) or vehicle (0.9% NaCl) at 1 and 2 h post-TBI. Extracellular field potentials (fEPSP) to Schaffer Collateral stimulation were recorded in CA1 at 1 and 3 days following treatment. (**A**) LTP was recorded in both the contralateral (Contra-Hip) and ipsilateral (Ipsi-Hip) hippocampal slices at 1 d (**A1**) and 3 d (**A2**) following vehicle (left) and CN2097 treatment (right). Left: Following TBI, LTP (2 × 100 Hz for 1 s, 20 s apart; arrows) was impaired in the ipsilateral (Ipsi-Hip, red), but not the contralateral hippocampus (Contra-Hip, black). Right: CN2097 restored LTP to control levels. Field EPSP slopes in the CA1 region are plotted as a percentage of baseline. Insets: Examples of fEPSP traces representing averages of 20 responses at baseline and LTP (Contra, black; Ipsi, red). (**B**) Group data of LTP in vehicle- and CN2097-injected rats at 1 d (**B1**) and 3 d (**B2**) following TBI. (**B1**) At 1 d after TBI and vehicle treatment, LTP was significantly impaired in the ipsilateral compared to contralateral hippocampus. After CN2097 treatment, LTP was restored in the ipsilateral hippocampus to a level that was not significantly different from that observed in the contralateral hippocampus. (**B2**) At 3 d following TBI, synaptic plasticity was similarly restored. In CN2097-treated rats, LTP in the ipsilateral hippocampus was not different from that observed in the contralateral hippocampus, while vehicle treatment resulted in a significant interhemisphere difference (**C**). Interhemisphere ratio (Ipsi/Contra) of LTP indicates highly impaired LTP in the ipsilateral hemisphere of vehicle-treated rats (yellow) when compared to CN2097-treated rats (blue) at 1 d as well as at 3 d post-TBI. Data represent mean ± SEM.

As discussed above, an increase in JNK activity can disrupt LTP and hippocampal-dependent learning following TBI^22^. Therefore, we examined whether CN2097 could attenuate TBI-induced deficits in LTP. Long-term potentiation has been reported to be impaired in the CA1 area of the hippocampus at 2–3 days post-injury^30–32^, whereas others did not observe an LTP deficit^27^. Using the CCI model of TBI, we examined the capacity for LTP induction in the ipsilateral and contralateral hemispheres. Rats received intraperitoneal (i.p.) injections of vehicle at 1 and 2 h post-TBI. Hippocampal slices were prepared at 1 and 3 days after CCI injury for extracellular recordings. Extracellular field potentials (fEPSPs) evoked at hippocampal CA1 pyramidal cells by the electrical stimulation of the Schaffer collateral-commissural pathway were recorded at 0.33 Hz. The stimulation intensity used was adjusted to the intensity generating half-maximal response amplitudes in both hemispheres. After recording a stable baseline for more than 20 min, LTP was induced by 2 sets of high frequency stimulation (HFS, 2 × 100 Hz for 1 s, 20 s apart). As shown in Fig. 3, rats receiving vehicle exhibited deficits in LTP in the ipsilateral (Ipsi-Hip) relative to the contralateral (Contra-Hip) hippocampal slice at 1 d (Fig. 3A 1) and 3 d (Fig. 3A2) after injury. In the ipsilateral hippocampus, LTP was significantly impaired (104.6 ± 2.3%, n = 8) compared to the contralateral hippocampus (158.9 ± 5.4, n = 7, p < 0.0005; Ipsi/Contra ratio = 0.66) (Fig. 3B1) at 1 d after TBI and vehicle treatment. A similar interhemisphere difference (Ipsi: 106.3 ± 3.4, n = 12, Contra: 157.0 ± 7.1, n = 11, p < 0.0005; Ipsi/Contra ratio = 0.68) was observed 3 d after vehicle treatment (Fig. 3A2, left; 3B2, left).

Next we tested whether CN2097 could ameliorate the LTP deficits caused by neurotrauma. Rats were injected i.p. with CN2097 at 1 and 2 h post-TBI. At 1 d after TBI combined with CN2097 treatment, LTP was restored in the ipsilateral hippocampus (142.6 ± 2.5, n = 7) to a level that was not significantly different from that observed in the contralateral hippocampus (141.6 ± 10.9, n = 5, p = 0.91; Ipsi/Contra ratio = 1.01) (Fig. 3A 1 and B1, right). Similarly, at 3 d following CN2097 treatment, LTP was not significantly different in the ipsilateral (155.8 ± 6.4, n = 10) versus the contralateral hippocampus (160.1 ± 8.8, n = 6, p = 0.87; Ipsi/Contra ratio = 0.97) (Fig. 3A 2 and B2, right). Interhemisphere ratios (Ipsi/Contra) of LTP for all treatment groups summarized in Fig. 3C suggest that CN2097 injected 1 and 2 h following TBI can restore LTP at 1 and 3 days after injury.

**Figure 4:**
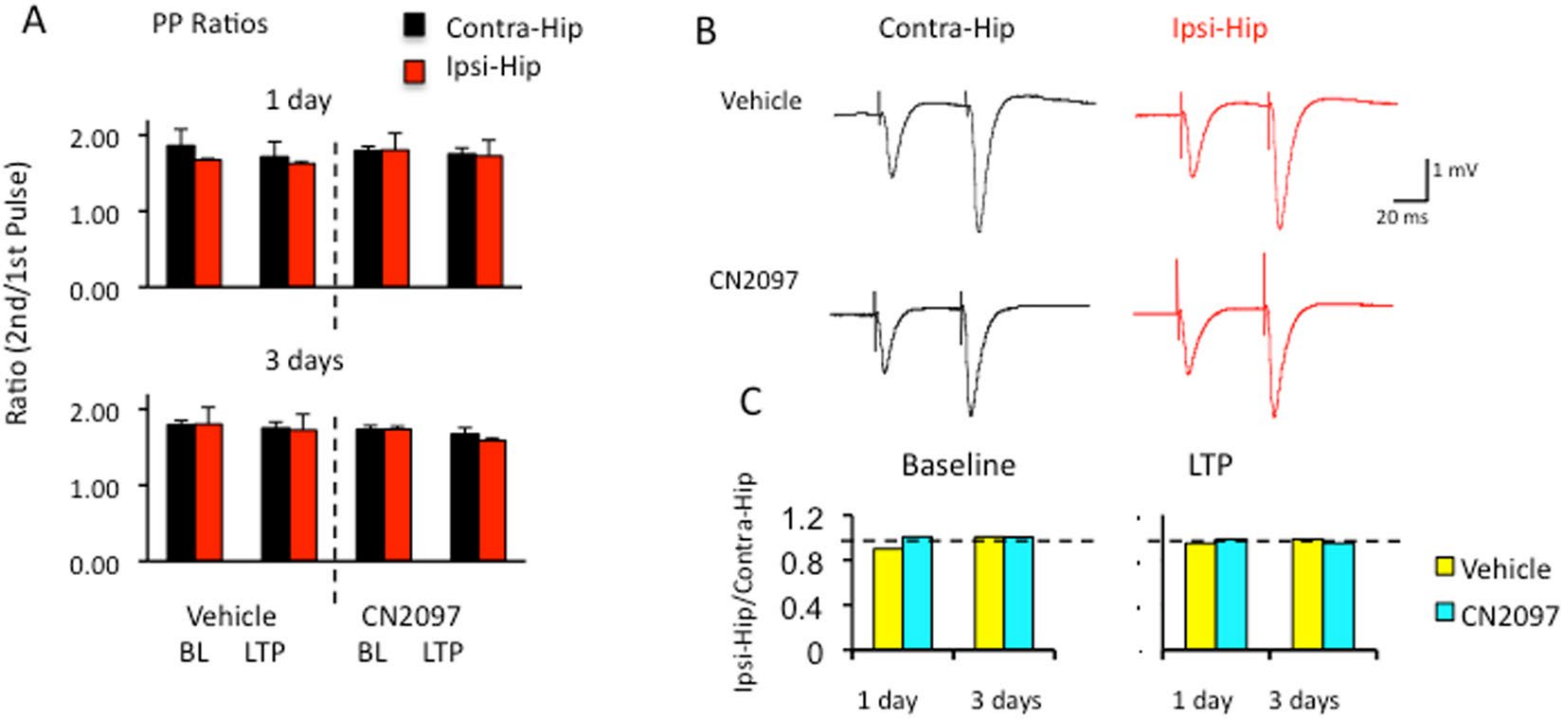
Presynaptic mechanisms are not involved in TBI-dependent LTP impairment and CN2097-induced LTP restoration. (**A**) Paired pulse ratios (PPRs) for vehicle- and CN2097-treated rats in both hemispheres at 1d (top) and 3 d (bottom) after TBI at baseline (BL) and after LTP induction (LTP). (**B**) Examples of fEPSP traces representing averages of 10 responses at baseline in the contralateral (Contra-Hip, black) and ipsilateral (Ipsi-Hip, red) hippocampi in vehicle- (top) and CN2097-treated (bottom) rats at 3 d following TBI. Results indicate no differences between groups. (**C**) Interhemisphere ratios (Ipsi/Contra) of PPR results at baseline (left) and after LTP induction (right) for vehicle- (yellow) and CN2097-treated (blue) rats. Neither TBI nor CN2097 treatment modifies PPRs. Data represent mean ± SEM.

To explore whether presynaptic transmitter release is involved in the suppressed LTP caused by TBI, we stimulated hippocampal slices with two pulses separated by 50 ms. The probability of transmitter release can be quantified by the amplitude ratio of the second to the first pulse. As shown in Fig. 4A, we observed no difference in the paired pulse ratio (PPR) between ipsilateral and contralateral hemispheres at 1 d (Ipsi: BL 1.67 ± 0.02, LTP 1.63 ± 0.03, n = 12; Contra: BL 1.86 ± 0.22, LTP 1.71 ± 0.20, n = 8) and 3 d (Ipsi: BL 1.54 ± 0.05, LTP 1.40 ± 0.06, n = 13; Contra: BL 1.62 ± 0.02, LTP 1.55 ± 0.03, n = 13) after TBI and vehicle injection. Similar results were obtained at 1 and 3 days in rats that received CN2097 injections at 1 and 2 h following TBI (1 d: Ipsi: BL 1.80 ± 0.022, LTP 1.72 ± 0.21, n = 8; Contra: BL 1.79 ± 0.06, LTP 1.75 ± 0.08, n = 5; 3 d: Ipsi: BL 1.74 ± 0.04, LTP 1.59 ± 0.03, n = 10; Contra: BL 1.73 ± 0.06, LTP 1.67 ± 0.09, n = 6) (Fig. 4A). Interhemisphere ratios (Ipsi/Contra) were not different at 1 and 3 days whether rats were treated with vehicle (0.9 and 0.95, respectively) or CN2097 (1.01 and 1.00, respectively) at baseline (Fig. 4C, left) or after LTP (vehicle: 0.95 and 0.91, respectively; CN2097: 0.99 and 0.95, respectively; Fig. 4C, right). These results suggest a non-presynaptic mechanism to be involved in TBI-induced LTP deficits and LTP restoration in the presence of CN2097.

### The effect of CN2097 on neurobehavioral outcomes after TBI

**Figure 5:**
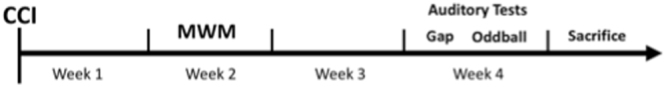
Timeline for behavioral studies including relative dose timing and weeks of MWM and auditory testing.

**Figure 6:**
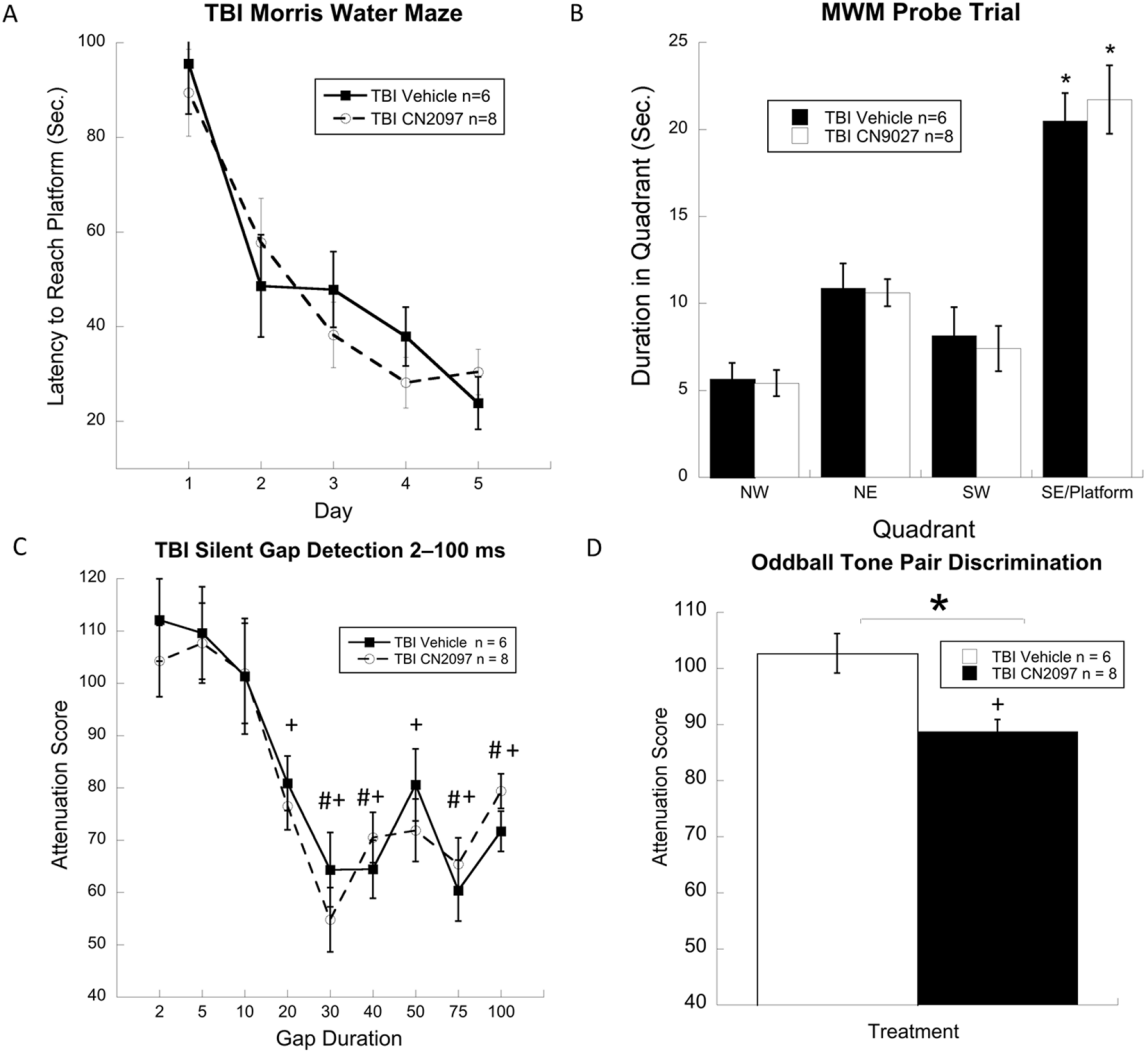
Morris water maze and probe trial performance, and auditory sensory outcome after TBI. The line graph (**A**) shows a significant reduction in latency to reach the hidden platform for injured rats treated with CN2097 at a dose of 10 mg/kg injected i.p. at 1 and 2 h post-TBI (TBI CN2097; n = 8) and vehicle (0.9% NaCl) (TBI Vehicle; n = 6). The bar graph (**B**) shows that both groups spent significantly more time in the platform quadrant when compared to non-platform quadrants, suggesting comparable memory performance in both groups for the former platform location during the probe trial. Graphs also show (**C**) comparable basic auditory temporal processing of silent gaps in white noise for both groups and (**D**) a significant improvement of complex tone order discrimination in rats treated with CN2097 versus those receiving vehicle (*p = 0.004). ^+^Indicates significant (p < 0.05) difference between cued and uncued responses for CN2097-treated rats. ^#^Indicates significant (p < 0.05) difference between cued and uncued responses for vehicle-treated rats. Data represent mean ± SEM.

We evaluated the efficacy of CN2097 on behavioral tasks related to spatial learning and sensory processing, deficits of which have been observed in TBI patients^33, 34^. A timeline for these behavioral studies, including relative dose timing and weeks of Morris water maze (MWM) and auditory testing, is shown in Fig. 5. Hippocampal-dependent spatial learning is vulnerable to brain injury^35^ and can result in the inability to navigate a novel environment^33^. To determine if CN2097 treatment after TBI impacts spatial learning and memory, rats underwent the MWM testing for 5 d, followed by a single probe trial. Results from 2 treatments (TBI-vehicle, n = 6, and TBI-CN2097, n = 8) by 5 d repeated measures ANOVA showed that both groups performed similarly across all days of testing with all rats significantly improving from day one to day five (F(4,48) = 25.2, p < 0.001) (Fig. 6A). Similarly, results from the probe trial (Fig. 6B), a final swim in which the platform was removed, indicated that both treatment groups spent more time in the former platform zone, when compared to the non-platform zones, indicating intact memory for the former platform location in both groups (F(3,36) = 41.0, p < 0.01).

Central auditory processing deficits have been reported in patients after TBI^34^, and, similar to those in human TBI studies, auditory assessments have been used to identify impairments in basic and/or more complex auditory processing in rodent models of brain injury^36–38^. To assess auditory temporal processing, rats were tested for auditory gap detection. For CN2097-treated rats, paired samples *t*-tests revealed significant differences between cued and uncued peak response scores at the gap durations of 20 (p < 0.001), 30 (p < 0.01), 40 (p < 0.01), 50 (p < 0.01), 75 (p < 0.01), and 100 ms (p < 0.01) (Fig. 6C), indicating significant detection of these cue durations. In comparison, for vehicle-treated rats, paired samples *t*-tests revealed significant differences between cued and uncued peak response scores at 30 (p < 0.05), 40 (p < 0.05), 75 (p < 0.05), and 100 ms (p < 0.05) (Fig. 6C), indicating significant detection at fewer gap durations in contrast to CN2097-treated rats. However, results from 2 treatments (TBI-vehicle, n = 6, and TBI-CN2097, n = 8) by 9 gaps (2–100 ms) repeated measures ANOVA revealed no effect of treatment (F(1,12) = 0.05, p = 0.83) (Fig. 6C). In addition, a significant effect of gap duration (F(1,12) = 93.9, p < 0.001) indicated that as gap duration increased, detection for both treatment groups improved in a similar manner. In contrast, when both groups were presented with a more complex tone order discrimination task (Fig. 6D), results from an independent samples *t*-test revealed a significant effect of Treatment (*t*(1,13) = 12.7, p = 0.004), with CN2097-treated rats showing superior detection when compared to vehicle-treated animals. In this complex task, a paired samples *t*-test revealed significant differences between cued and uncued peak responses for CN2097-treated animals (p < 0.01), indicating significant detection of the tone reversal. In contrast, vehicle-treated rats showed no difference between cued and uncued peak responses (p = 0.43), indicating an inability to detect the tone pair reversal. These findings suggest that TBI differentially affects spatial learning and auditory processing domains and that treatment with CN2097 can improve complex auditory processing after injury.

### The effect of CN2097 on post-traumatic loss of neural tissue

**Figure 7:**
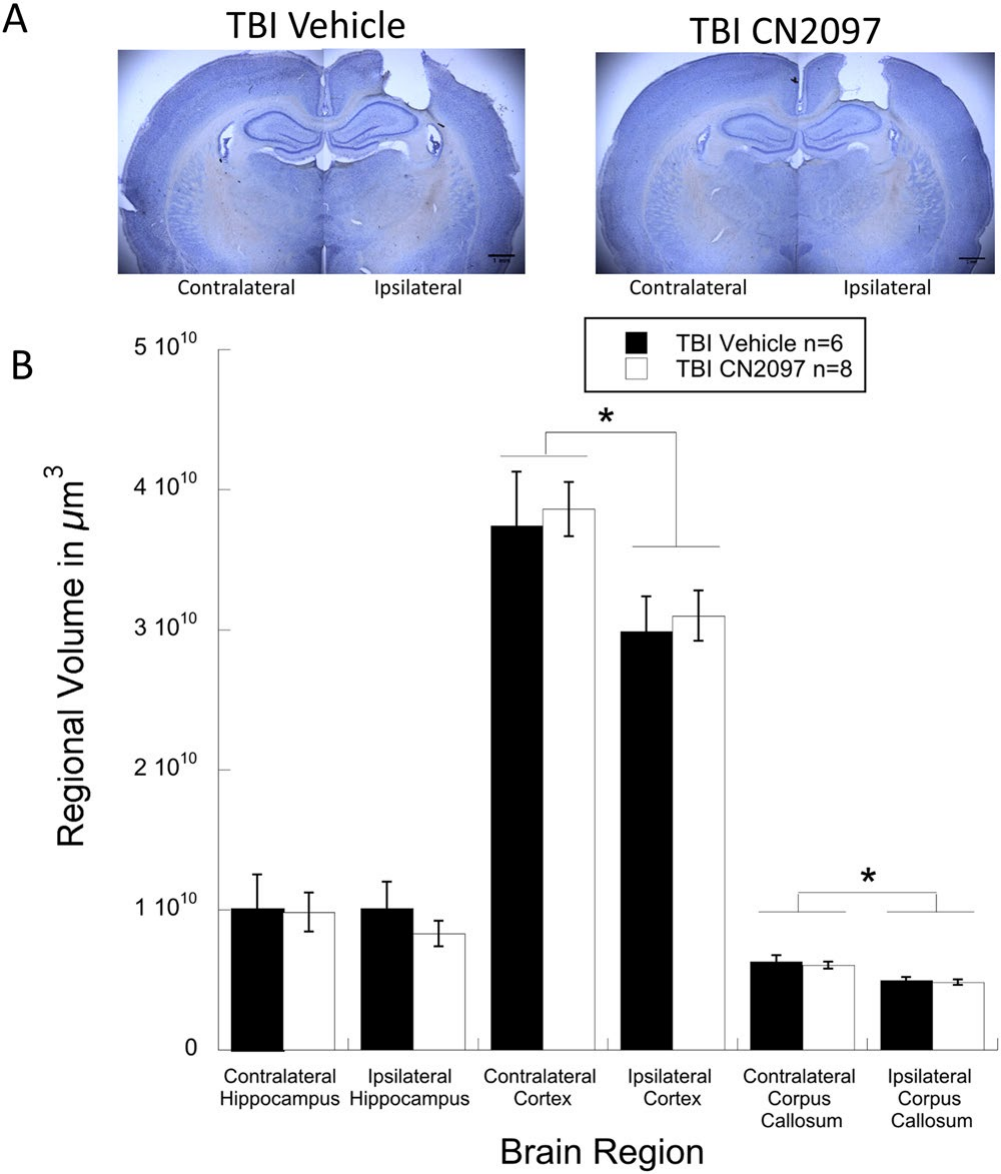
Brain regional volume analysis in CN2097- versus vehicle-treated rats. Photomicrographs (**A**) show coronal brain sections from ipsilateral and contralateral hemispheres from vehicle- and CN2097-treated rats, demonstrating comparable injury profiles. Bar graph (**B**) shows regional brain volume measurements for vehicle-treated (TBI Vehicle; n = 6) and CN2097-treated (TBI CN2097; n = 8) rats, indicating comparable ipsilateral and contralateral regional brain volumes of the cerebral cortex, hippocampus, and corpus callosum. *Indicates significant (p < 0.01) differences between ipsilateral and contralateral hemispheres for both treatment groups. Data represent mean ± SEM.

Regional volume analysis of 14 brains (vehicle-treated rats, n = 6, and CN2097-treated rats, n = 8) was performed after the completion of behavioral tests. This analysis included the ipsilateral and contralateral cerebral cortices, hippocampi, and the corpus callosum. For vehicle-treated rats, repeated measures ANOVA for 2 hemispheres (ipsilateral and contralateral) by 3 brain regions (cerebral cortex, hippocampus, and corpus callosum) revealed a significant main effect of hemisphere (F(1,10) = 11, p < 0.05), indicating that the volume of the ipsilateral hemisphere was significantly smaller compared to the contralateral hemisphere. Paired samples *t*-tests revealed significant differences between the volumes of ipsilateral and contralateral cerebral cortices (p < 0.01) and the corpus callosum (p < 0.01) in this group of rats (Fig. 7). Similarly, for CN2097-treated animals, repeated measures ANOVA for 2 hemispheres by 3 brain regions (as described above) revealed a significant main effect of hemisphere (F(1,14) = 39.8, p < 0.05), indicating that the structures in the ipsilateral hemisphere were significantly smaller than those in the contralateral hemisphere. As with vehicle-treated rats, the paired samples *t*-tests revealed that in CN2097-treated animals, main effects were a product of significant reductions in volumes of cerebral cortices (p < 0.001) and corpus callosum (p < 0.001) ipsilateral to injury (Fig. 7). No significant differences were observed between ipsilateral and contralateral hippocampi in two treatment groups. These findings indicate that the TBI model used in this study produces gross structural reductions in various brain areas ipsilateral to injury. Further analysis involving independent samples *t*-tests for the above-described brain areas across the treatment groups showed no effect of treatment on the volumes of these areas (Fig. 7). These results indicate that with the CN2097 dose used the improvements seen in other measures in CN2097-treated animals do not extend to the gross structural level.

## Discussion

Several pathophysiological processes triggered by neurotrauma, including glutamate excitotoxicity, oxidative stress, and neuroinflammation, are initiated within the first hours after TBI, but result in prolonged neurodegeneration^39^. At synapses, the activation of NMDARs has been shown to promote the release of BDNF^40^ required for survival^3^ and cognition^6, 12^. Although numerous studies have shown the neuroprotective potential of BDNF, treatment with BDNF is limited by its short plasma half-life and poor BBB penetration^41^. Here, using the CCI rat model of TBI, we demonstrate the therapeutic efficacy of CN2097, a novel cyclic peptide that enhances synaptic BDNF signaling^12^ and blocks NMDAR-mediated cell-death signaling^13^.

Therapeutic interventions aimed at reducing post-traumatic neuroinflammation and limiting the influx of inflammatory cells diminish the loss of neural tissue and improve functional outcome after brain injury^39, 42, 43^. Here we report that post-injury treatment with CN2097 significantly attenuates JNK signaling, lowers the synthesis of proinflammatory cytokines, TNF-α and IL-1β, and diminishes the influx of inflammatory cells into the injured brain parenchyma. Although the cellular mechanisms underlying the anti-inflammatory activity of CN2097 are poorly understood, it appears that CN2097 interferes with the initiation/progression of inflammatory cascade. Similarly, the TrkB agonist, 7,8-dihydroxyflavone, has been reported to decrease microglial release of IL-1β and TNF-α by downregulating MAPK signaling^24^. CN2097 may also improve functional outcome due to its inhibitory effect on the post-traumatic synthesis of MMP9. This conclusion is supported by previous observations that the genetic or pharmacological interference with MMP9 synthesis reduces neuronal loss and improves neurobehavioral performance after injury^44^.

Neuroinflammation encompasses a broad range of pathophysiological phenomena, which include the production of soluble proinflammatory mediators, the invasion of inflammatory cells, and the activation of microglia. A significant increase in brain production of proinflammatory cytokines and chemokines occurs within hours after TBI, but usually lasts for a relatively short period of time, less than 24 h, after injury^45^. Similarly, the post-traumatic invasion of neutrophils is observed in the early stage post-TBI; in comparison, monocytes infiltrate the traumatized parenchyma within days after TBI. These phenomena coincide temporally with a peak in neuronal death resulting from TBI^46^, suggesting that anti-inflammatory intervention initiated shortly after TBI is critical for attenuating the post-traumatic loss of neural tissue. Therefore, an early, single dose of a drug mitigating post-traumatic neuroinflammation could be therapeutically efficacious. This idea is not only supported by the present results, but also by the data from other laboratories^47, 48^. While post-traumatic neuroinflammation is also characterized by a long-lasting activation of microglia^49^, it is presently unclear what role these glial cells might play in the chronic phase after injury and how to approach this therapeutically. It has been shown, for example, that microglia may have a neuroprotective role after brain injury^50^, which suggests that more research is needed to better understand this aspect of post-traumatic neuroinflammation.

In agreement with previous studies^5, 30, 31^, LTP was impaired in the ipsilateral relative to the contralateral hippocampus 1 and 3 days after injury, and CN2097 was found to attenuate these LTP deficits. We observed no difference in paired-pulse facilitation (PPF), a general index of excitation-neurotransmitter release coupling. BDNF plays a key role in synaptic transmission, and proinflammatory cytokines, such as IL-1β, suppress TrkB-induced signaling required for LTP stabilization^16^. The ability of CN2097 to enhance TrkB-mediated PLCγ and the PI3K-Akt activity^12^, required for the induction^28^ and late-phase LTP^51^, may play a role in post-TBI recovery of hippocampal LTP^52^. Cytokines are reported to impair LTP via the JNK pathway activation^22, 29^, and we observed a reduction in JNK signaling in the hippocampus of CN2097-treated animals. An improvement in LTP performance and functional outcome in CN2097- versus vehicle-treated rats is also consistent with the ability of this compound to attenuate post-traumatic neuroinflammation.

Sensory cortices are highly susceptible to experience-dependent plasticity relying on LTP-dependent mechanisms as well as trauma-induced reorganization^53, 54^. This study shows that while the basic auditory discrimination appears to be intact in rats sustaining TBI, complex tone pair discrimination is impaired after injury. Thus, when rats were presented with a cortically mediated oddball tone order discrimination task at 4 weeks after injury, CN2097-treated rats performed significantly better than those receiving vehicle. Although we did not include a sham-injured group in this comparison, results show that only the CN2097-treated rats were able to significantly detect the complex tone reversal, reinforcing the significant treatment effect. These findings parallel reports of impaired event-related potential activity and central auditory acuity in TBI patients^34, 55–57^.

As CN2097 was found to attenuate hippocampal LTP deficits at 1 and 3 days after injury, we examined if CN2097 treatment could have an impact on spatial learning and memory. To this end, we employed the MWM paradigm, which has been used to assess cognitive deficits in TBI models^54, 58^. Results showed that both the vehicle- and CN2097-treated TBI groups performed similarly across all days of testing. The learning and memory profiles of both groups in the present study are comparable to those reported for control animals from other studies, suggesting that the severity of TBI might not have been sufficient to reach a threshold for behavioral impairment on the MWM task^35, 58, 59^. Moser and colleagues showed that direct lesions of dorsal hippocampus have to exceed 20% of the hippocampal tissue to consistently affect spatial learning, whereas the comparable lesions of the ventral hippocampus have no effect on performance in the MWM^60^. The disassociation between spatial learning and auditory discrimination observed in the current study suggests that tone-order discrimination may be a more sensitive measure of treatment efficacy in experimental TBI compared to MWM. The significance of our findings cannot be overstated as our relatively novel behavioral tests provide a highly sensitive means of assessing higher order sensory processes in the rodent model, which are adversely affected in TBI patients. Increasingly, these sensory processing tools have been employed in human clinical settings as outcome assessment methods given the critical role of auditory sensory discrimination in mediating normal speech function^34, 61^. These methods have also been used to evaluate models of developmental brain injury, which parallel sensory processing deficits seen in human language learning impaired populations^62^. The acoustic discrimination methods employed in the current study are highly sensitive to subtle cortical disruption^34^, and are translatable into clinical practice.

In this study, we showed that post-traumatic treatment with CN2097 (injected i.p. at a dose of 10 mg/kg at 1 and 2 h after injury) improves synaptic plasticity and behavioral outcome; however, CN2097 administered at this dose did not appear to reduce the volume of post-traumatic lesion. The 10-mg/kg dose of CN2097 used was based on the intravitreal dosing of this peptide to attenuate NMDA-induced poly-ADP-ribosylation in the retina in an *in vivo* model of retinal toxicity^13^, and the dosing of intrathecally injected CN2097 to define its central anti-nociceptive effects^63^. In future studies, it will be necessary to assess the optimal dose of this cyclic peptide that could also reduce the neuronal loss resulting from injury. The rationale for beginning treatment at 1 h post-TBI was based on the reasonable assumption that within this time frame after injury the patient could be treated at the scene or shortly after arriving at the hospital. Since the patient may not always be available for therapeutic intervention at such early time after TBI, it will also be important to define the potential therapeutic time-window for delayed treatment with CN2097.

Traumatic brain injury remains a major health and socioeconomic problem. As the severity of secondary injury largely determines functional outcome after TBI, early intervention aimed at mitigating secondary injury is critical for improving long-term neurological outcome. The complex and interdependent nature of pathophysiological events resulting from TBI may explain why single-mechanism neuroprotective interventions have failed to demonstrate consistent improvement of outcome in neurotrauma patients^2^. Our results suggest that the development of multifunctional drugs, such as CN2097, that simultaneously reduce neuroinflammation and facilitate synaptic plasticity, will have significant therapeutic potential to promote functional recovery after TBI.

## Materials and Methods

### Controlled cortical impact injury

Adult male Long-Evans weighing 200–250 g (Harlan, Indianapolis, IN) were kept at 22 °C with a 12-h light cycle and maintained on standard pellet rat chow and water *ad libitum*. The surgical and animal care procedures used in this study were approved by the Animal Care and Use Committee of Rhode Island Hospital and conformed to international guidelines on the ethical use of animals. All experiments were performed in accordance with these guidelines and regulations. Four to six rats per group/time point were used for most experiments with the exception of behavioral studies in which 6–8 animals was tested. The controlled cortical impact (CCI) model of TBI was employed as previously described^14, 26, 64^. In brief, rats were anesthetized with i.p. pentobarbital sodium (60 mg/kg) and a 4-mm craniotomy was performed on the right side of the skull to expose the dura, with the center of the opening located 3 mm posterior to the bregma and 2.5 mm lateral to the midline. The velocity of impact was 5 m/sec and the duration of impact was 50 msec. The diameter of the impactor tip was 2.5 mm and the depth of brain deformation was set at 2.5–3.0 mm. For all types of experiments, animals were injected i.p. with CN2097 (10 mg/kg) or vehicle (0.9% NaCl) at 1 and 2 h post-TBI. For Western blot analysis, animals (n = 4 rats/group) were sacrificed at 4 h or 24 h after TBI and the cerebral cortical or hippocampal samples were collected. The effect of CN2097 on hippocampal LTP was evaluated at 1 and 3 days post-TBI. The effect of CN2097 on neurobehavioral outcome after TBI was tested as shown in Fig. 5.

### Western blotting

Proteins from brain tissue samples were extracted using RIPA buffer (150 mM NaCl, 50 mM Tris-HCl, pH 7.4, 2 mM EDTA, 1% Triton X-100, 0.5% deoxycholate, 0.1% SDS), containing protease inhibitors (1 mM benzamidine, 100 U/ml aprotinin, 20 μg/ml antipain, 20 μg/ml leupeptin, 1 μg/ml pepstatin A, 1 mM PMSF) and phosphatase inhibitors (10 mM sodium pyrophosphate, 1 mM sodium orthovanadate, 1 mM sodium fluoride, 1 mM β-glycerophosphate). Proteins were resolved via SDS-polyacrylamide gel (4–12%) electrophoresis under reducing conditions and were transferred onto 0.2-μm nitrocellulose membranes (Invitrogen, Carlsbad, CA). After blocking with 5% ECL Advance blocking agent (GE Healthcare, Little Chalfont, UK) for 1 h at room temperature, the membranes were incubated with primary antibodies overnight at 4 °C. The following antibodies were used: rabbit polyclonal anti-human TNF-α (diluted 1:500) and anti-human IL-1β (4 µg/mL) from Novus Biologicals (Littleton, CO); rabbit monoclonal anti-human matrix metalloproteinase 9 (MMP9) (0.4 µg/mL) from Abcam (Cambridge, MA); mouse monoclonal anti-rat CD68 (clone ED1; 0.5 µg/mL) from Serotec (Oxford, UK) and anti-chicken α-tubulin (clone DM1A; diluted 1:5000), JNK and p-JNK (Thr183/Tyr185) from Cell Signaling (Danvers, MA). Membranes were subsequently incubated with horseradish peroxidase-conjugated anti-rabbit or anti-mouse antibody (Cell Signaling; diluted 1:5000) for 1 h at room temperature. Detection was performed using Lumigen TMA-6 (Lucien, Southfield, MI) or ECL Prime (GE Healthcare) chemiluminescence detection reagents. The Bio Imaging System Chemo Genius2 (Syngene, Frederick, MD) was used for Fig. 1 and the Bio-Rad ChemiDoc XRS+ system (chemi hi res and exposure time of 17–20 seconds) for Fig. 2. In the analysis of the optical density of the bands on immunoblots, the levels of proteins of interest were normalized to the levels of α-tubulin. The analysis of optical density was performed using ImageJ software (http://rsb.info.nih.gov/ij/).

### *In vitro* slice preparation

Rat brains were immersed in cold (5–7 °C), oxygenated (95% O_2_/5% CO_2_) artificial cerebrospinal fluid (ACSF) containing (in mM): 126 NaCl, 3 KCl, 1.25 NaH_2_PO_4_, 1 MgSO_4_, 2 CaCl_2_, 26 NaHCO_3_, 10 glucose. Coronal slices (500 µm), including the dorsal hippocampus and adjoining cerebral cortex, were cut using a vibratome. Slices were transferred to a temperature controlled (34 ± 0.5 °C) interface chamber and superfused with oxygenated ACSF at a rate of 1–2 mL/min. Slices were allowed to recover for at least one hour prior to the start of recordings.

### Stimulation and field potential recordings

Animal procedures were performed in compliance with the US Department of Health and Human Services and the IACUC animal care guidelines at Brown University. Recording electrodes pulled from borosilicate glass pipettes (resistance < 1MΩ) were placed within the CA1 stratum radiatum for extracellular recordings. Synaptic responses were elicited by stimulation of the Schaffer collaterals with square wave pulses using concentric bipolar stimulation electrodes. To assess paired-pulse ratio, which is a measure for presynaptic release probability, each stimulation consisted of two pulses separated by 50 ms. Extracellular postsynaptic field potentials were recorded using an AxoClamp2B amplifier (Axon instruments) and EX1 differential amplifier (Dagan), and digitized at 10 kHz. Data was acquired using Igor Pro (Wave Metrics) and Neuromatic (www.neuromatic.thinkrandom.com). For LTP induction, the stimulus intensity eliciting 50% of the maximum amplitude was used for all measurements before and after LTP induction. Baseline amplitudes were recorded for 20–30 minutes using single stimuli applied every 30 sec. Following a stable baseline period, LTP was induced by two sets of high-frequency stimulation (HFS) at 100 Hz for 1 sec, 20 sec apart. Extracellular postsynaptic field potential slopes were measured and LTP values were expressed as percentage of mean baseline ± SEM. Paired two-tailed *t*-tests were used for statistical analysis. For PPR analysis, ratios of the 2nd to the 1st pulse were assessed and compared between treatment groups. Because the uninjured contralateral hemispheres exhibited the same magnitude of LTP in vehicle- and CN2097-treated rats, we used the contralateral hemisphere as a control for treatment-dependent effects. We then calculated the ratios between the contralateral and ipsilateral hemispheres to assess the ability of CN2097 to improve LTP in the ipsilateral hippocampus in which it was impaired by injury.

### Morris water maze

Briefly, the MWM testing was conducted one week following CCI in a round tub 122 cm in diameter filled with water at 22 °C with a 20-cm diameter transparent plexiglass submerged platform, consistently placed in the southeast (SE) quadrant, 2 cm below the water surface (see Fig. 5 for timeline). Fixed, extra-maze cues included posters with geometric shapes on adjacent walls. On each of five testing days, rats underwent four trials, with each trial starting from a different randomly selected compass point (N, S, E, W). On day one, trial one, each rat was placed on the platform for 10 s, removed from the platform and then released from one of the starting locations. Each trial was limited to a maximum duration of 45 s. Animals unable to reach the platform within this time were guided to the target and remained there for 5 s. The latency to reach the platform for each trial across days was recorded using Ethovision XT (Noldus) behavioral tracking software. Total time to reach the platform for each rat was summed across the four trials each day and used as the dependent variables. On day six, all rats were given a single 45 s probe trial, where the platform was removed and the time spent in the four quadrants was recorded (NW, NE, SW, SE/Platform quadrants). Greater time spent in the former platform zone is indicative of memory for the platform location.

### Auditory discrimination

Auditory testing took place during the fourth week post-TBI (see Fig. 5 for timeline) and involved a modified acoustic startle paradigm that has been discussed extensively elsewhere^36, 65, 66^. Two tasks were presented (gap detection and odd-ball tone pair discrimination), which have been widely used to assess auditory temporal processing in humans and rodent models^67, 68^. Briefly, the startle modification paradigm involved the presentation of an auditory cue prior to a startle-eliciting stimulus (SES). The SES elicits an acoustic startle reflex (ASR) and if the preceding auditory cue is detected, the intensity of the ASR is reduced. Stimulus files were played through a Pyle PT8000CH amplifier connected to four 200-Watt PCB4 Pyle speakers (Pyle Audio inc., Brooklyn, NY), with sound levels calibrated by sound-level meter^66^. Attenuated response scores (ATT) were calculated from the peak ASR using the formula ([mean cued response/mean uncured response] × 100) and used as dependent measures. Thus, lower attenuation scores reflect greater relative detection of the cued stimuli. First, rats were given one day of testing on a gap detection task (0, 2, 5, 10, 20, 30, 40, 50, 75, or 100 ms). The gap session included 300 trials, each consisting of the presentation of variable duration silent gaps embedded in continuous 75-dB broadband white noise. Each silent gap was presented 50 ms prior to a 105-dB burst of white noise. During uncued trials, the 105-dB burst immediately followed the 75-dB white noise^65, 69^. Second, the oddball session was comprised of 104 trials. This procedure involved the repeated presentation of a background 75-dB, high-low frequency tone sequence (2300–1100 Hz) separated by a within-stimulus inter-stimulus interval (ISI) of 225 ms. Each sequence was separated by a between sequence ISI of 425 ms (200 ms greater than the inter-stimulus interval to maintain perceptual contiguity of the tone-pair). On uncured trials, the last tone sequence was followed by 50 ms of silence, then by the 105-dB/50 ms SES. On cued trials, a reversal of the tone sequence occurred (low-high; 1100–2300 Hz) followed by 50 ms of silence, and then the SES.

### The assessment of changes in brain regional volumes

After behavioral testing, rats were transcardially perfused with 200 mL of 0.9% NaCl solution and 200 mL of 10% phosphate-buffered formalin. Each brain was sectioned using a vibrating microtome at 100 µm in the coronal plane. Every section from approximately –1.0 to –4.2 mm from bregma was mounted on glass slides, stained using a standard cresyl violet protocol, and cover slipped in preparation for volume analysis. Regions of interests (cerebral cortex, hippocampus, and corpus callosum) were traced in Stereoinvestigator (MBF Bioscience, Williston, VT) and volume estimates were calculated in the same software program using Cavalieri’s unbiased estimator of volume derived from the serial section reconstruction^70^.

### Peptide synthesis

CN2097 was synthesized using *N*-(9-fluorenyl)methoxycarbonyl (Fmoc)-solid phase peptide synthesis protocols^20^, as previously described^13^. CN2097 was purified by RP-HPLC, lyophilized, and exchanged with HCl. Peptide purity was in the range of 90–95% as determined using high-resolution time of flight AXIMA-performance MALDI TOF-TOF mass spectrometer (Shimadzu).

## Electronic supplementary material

Supplementary information
