# Supplementary material for: TrkB-enhancer facilitates functional recovery after traumatic brain injury
Source: Sci Rep. 2017 Sep 8;7:10995. doi: 10.1038/s41598-017-11316-8 (PMC5591207; doi:10.1038/s41598-017-11316-8)
Supplement: Supplementary file 1 — Supplementary information [file 41598_2017_11316_MOESM1_ESM.pdf]

TrkB-enhancer facilitates functional recovery after traumatic brain injury

John Marshall[1], Joanna Szmydynger-Chodobska[2], Mengia S Rioult-Pedotti[1], Kara Lau[1], Andrea T. Chin[2], Siva K. Reddy Kotla[3], Rakesh Kumar Tiwari[3], Keykavous Parang[3], Steven W. Threlkeld[4] and Adam Chodobski[2]

[1]Department of Molecular Pharmacology, Physiology, and Biotechnology, Brown University, Providence, RI 02912, USA.

[2]Neurotrauma and Brain Barriers Research Laboratory, Department of Emergency Medicine, Alpert Medical School of Brown University, Providence, RI 02903, USA.

[3] Center for Targeted Drug Delivery, Department of Biomedical & Pharmaceutical Sciences, Chapman University School of Pharmacy, Irvine, CA 92618, USA.

[4]Department of Neuroscience, Regis College, Weston, MA 02493, USA.

To whom correspondence should be addressed: John Marshall, Ph.D. Email: John_Marshall@Brown.edu

Supplementary Figures and Figure legends

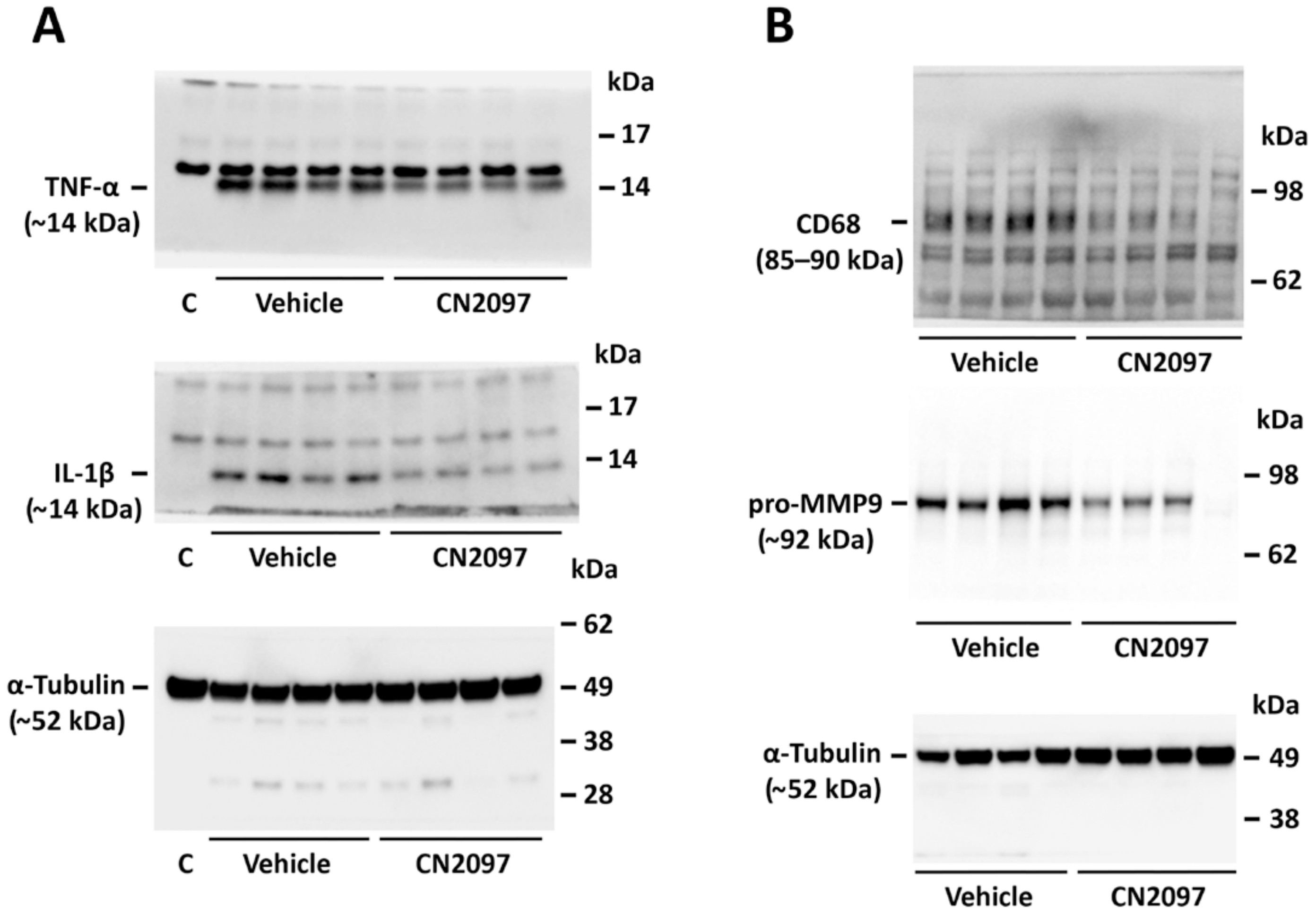

Figure S1. The original full-length Western blot images for Figure 1. Therapeutic efficacy of CN2097 in reducing post-traumatic neuroinflammation in the injured cerebral cortex.

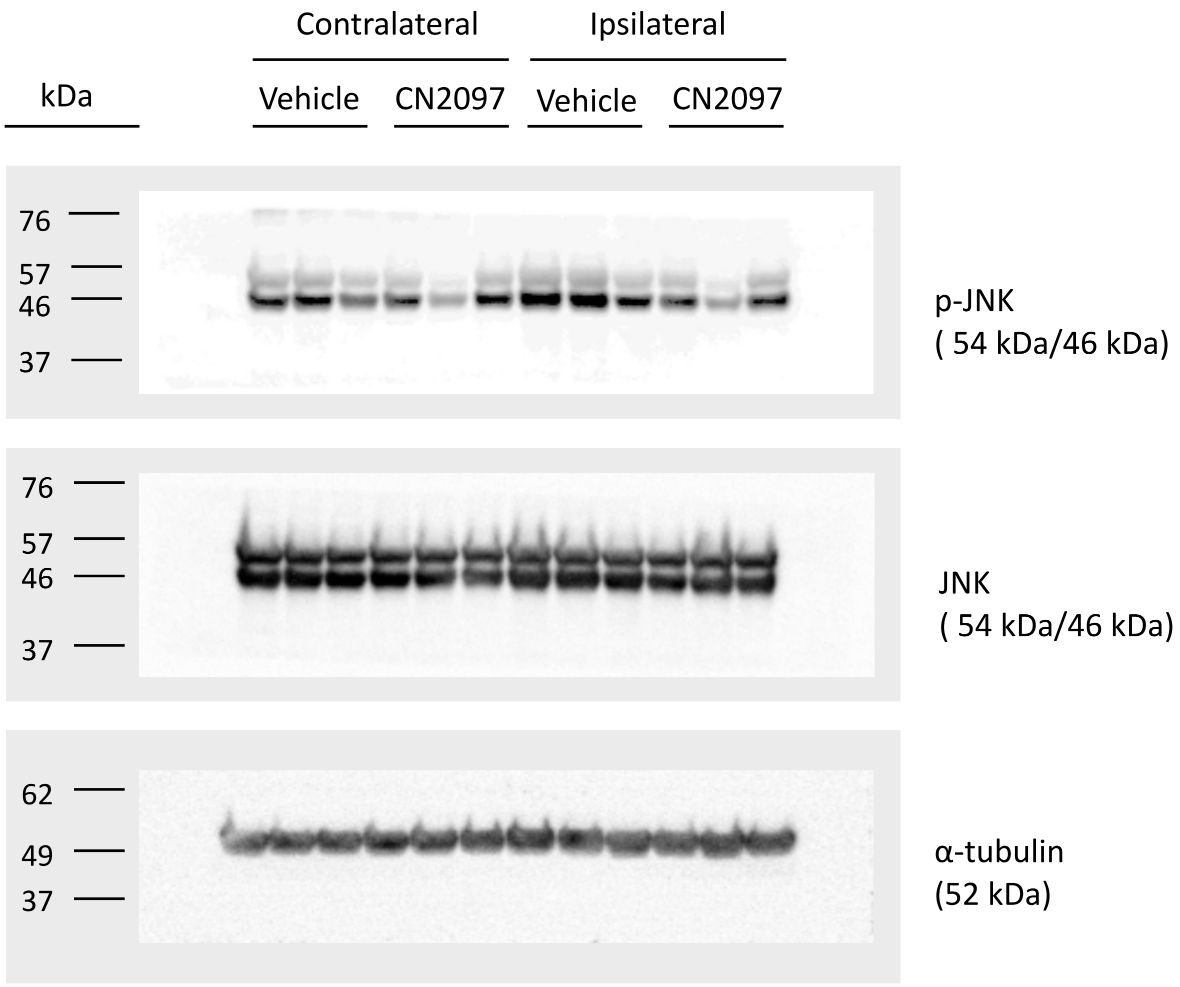

Figure S2. The original full-length Western blot images for Figure 2. CN2097 reduces JNK activity in the injured ipsilateral hippocampus.
